# Production of high quality and natural-like recombinant allergens needed for high efficiency of allergy treatment

**DOI:** 10.1186/2045-7022-4-S2-P7

**Published:** 2014-03-17

**Authors:** Veronique Gomord, Anne-Catherine Fitchette, Virginie Catala, Loïc Faye

**Affiliations:** 1 Angany Genetics, R&D, Val de Reuil, France

## Background

Due to a lack of sufficiently accurate and effective tools, allergy is often badly diagnosed. Moreover, its treatment is expensive, long, uncomfortable and of little effectiveness. In this context, less than a quarter of allergic patients receive a treatment currently. Allergies have therefore become an important public health issue, highlighting the many unmet medical needs. ANGANY Genetics has brought together expertise in protein genomics and in therapeutic protein production in plant expression systems, to produce recombinant allergens of an unprecedented quality, offering the following solutions to allergy sufferers: (1) a precise identification of each allergen involved rather than the current approximate diagnosis, (2) pure and certified recombinant allergens rather than imperfect and incomplete allergen extracts and (3) an effective treatment at the best cost to replace symptomatic-only or low-level curative allergy treatments.

## Method

In this context, ANGANY Genetics has developed AllergoPur™ for production of high quality natural-like recombinant allergens needed for high efficiency of allergy treatment. AllergoPur™ is a plant-based production platform using transient expression in N. benthamiana. This strategy offers a rapid, flexible and high-yielding production of recombinant allergens. Typically, high rates of recombinant allergens can be recovered twelve days after gene delivery. Furthermore, in contrast to bacterial cells, plants can produce recombinant allergens that require complex post-translational modifications and, obviously, plants are also the best system for expression of plant allergens requiring plant-specific maturations.

## Results

Using AllergoPur™, ANGANY Genetics has already produced high quality recombinant allergens from mites, pollens, mammals or mold. This is illustrated here with eight recombinant allergens required for an accurate diagnosis and an efficient immunotherapy of dust mite allergy. The detailed biological and structural characterizations of these recombinant allergens illustrate their quality and conformity to natural products.

## Conclusion

AllergoPur™ is a powerful tool for the rapid production of high quality natural like recombinant allergens. The capacity of this platform is illustrated with the successful expression of complex allergens impossible to produce in prokaryotic systems, such as Der p 1 or Hev b 13. Some of these allergens are currently tested in preclinical studies.

